# A multicenter, prospective, randomized controlled trial of intracranial hemorrhage risk of intensive statin therapy in patients with acute ischemic stroke combined with cerebral microbleeds (CHRISTMAS): Study protocol

**DOI:** 10.3389/fneur.2023.1097078

**Published:** 2023-02-09

**Authors:** Jia-ling Zhao, Chi-bo Ai, Li Wang, Shao-jie Yang, Jian Wang, Wei Yang, Jie Tang, Ling Zhang, Yan Li, Ting-qi Yan, Shu Gou, Gui-gui Xie, Yang Xiang

**Affiliations:** ^1^Department of Neurology, Sichuan Academy of Medical Sciences and Sichuan Provincial People's Hospital, Affiliated Hospital of University of Electronic Science and Technology of China, Chengdu, China; ^2^Department of Neurology, Yunyang County People's Hospital, Chongqing, China; ^3^Department of Neurology, Zigong Third People's Hospital, Zigong, China; ^4^Department of Neurology, Chengdu Eighth People's Hospital, Chengdu, China; ^5^Department of Neurology, Ya'an People's Hospital, Yaan, China

**Keywords:** acute ischemic stroke (AIS), cerebral microbleeds (CMBs), intracranial hemorrhage (ICH), statin, randomized controlled trial, protocol

## Abstract

**Background:**

Low serum levels of major lipid markers have been proved to be significantly associated with increased risks of hemorrhagic stroke (HS) and cerebral microbleeds (CMBs). However, there is no lipid modification guideline telling us how to maintain a balance between the prevention of ischemic stroke recurrence and the prevention of hemorrhagic events, especially in patients with acute ischemic stroke (AIS) and CMBs.

**Aim:**

The Intracranial **H**emorrhage **R**isk of Intensive **S**tatin **T**herapy in Patients with **A**cute **I**schemic **S**troke combined with **C**erebral **M**icrobleeds (**CHRISTMAS**) trial evaluates the risk of intracranial hemorrhage (i.e., HS and CMBs) of high-dose statin therapy in patients with AIS combined with CMBs.

**Methods and design:**

This is an investigator-initiated, multicenter, prospective, randomized controlled clinical trial design. Up to 344 eligible patients will be consecutively randomized to receive high-dose or low-dose atorvastatin in 1:1 ratio in 5 stroke centers in China.

**Outcomes:**

CHRISTMAS trial has co-primary outcomes, namely, hemorrhage risk: the incidence of HS and the changes in degree of CMBs until the end of 36-month follow-up.

**Discussion:**

The primary hypothesis of this study is that an excessive reduction in serum lipid levels by an intensive statin therapy in AIS patients with CMBs can increase the risk of intracranial hemorrhage. This study will shed light on new clinical decisions regarding the long-term serum lipid management in these patients with dilemma in clinical practice.

**Clinical trial registration:**

Clinicaltrials.gov, identifier: NCT05589454.

## Introduction

Cerebral microbleeds (CMBs) are a crucial radiological marker of cerebral small vessel disease (CSVD) (1) to illustrate the micropathology of perivascular hemosiderin deposition corresponding to past small foci of bleeding (2). The prevalence of CMBs increases with age and exceeds 20% in community population over 60 years old (3, 4). More importantly, CMBs are also a common comorbidity of stroke, brain trauma, Alzheimer's disease, and cerebral amyloid angiopathy (5, 6), and are associated with cognitive impairment, psychiatric symptoms, and decline of daily living ability (7).

CMBs have been identified to be indicative of bleeding-prone microangiopathy for their dynamical accumulation over time, with the burden (presence and number) of baseline CMBs predicting the development of new CMBs in both deep and lobar regions, and may predict spontaneous intracranial hemorrhage (ICH) (8–12). These pieces of evidence have suggested an important link between the presence of CMBs and future macrobleeding. However, CMBs have also been demonstrated in approximately one-third of patients with ischemic stroke (IS), that are associated with an increased risk of recurrent IS, symptomatic ICH and death (13). As the burden of CMBs in IS patients increased, the relative and absolute risk ratios for ICH increase steeply, although the absolute incidence of recurrent IS is consistently higher than that of ICH (13–16). Therefore, it raises clinical dilemmas, particularly regarding the safety of secondary prevention treatment of IS with concomitant presence of CMBs.

Statins have been widely used for secondary prevention of IS. The Stroke Prevention by Aggressive Reduction in Cholesterol Levels (SPARCL) study had proposed that the reduction in the risk of IS was primarily related to the extent to which low-density lipoprotein cholesterol (LDL-C) levels are lowered instead of statin use (17). It was the first to demonstrate that 80 mg of atorvastatin per day reduced the overall incidence of strokes and cardiovascular events in patients with recent stroke or transient ischemic stroke (TIA) and without known coronary heart disease (17). However, the correlation between statin use of an intensive dose and ICH risk has not been elucidated so far (18–20). A recent meta-analysis on intensive LDL-C-lowering statin-based therapy also revealed an increased risk of ICH related to high-dose statins (17, 21), while other studies did not show the similar conclusions that statin use (22) or intensive reduction of LDL-C (23) increased the ICH risk. More importantly, none of these previous studies have explored the relationship between intensive statin therapy and hemorrhage risk from the perspective of CMBs.

However, it is worth noting that low levels of serum triglycerides (TG), total cholesterol (TC), and LDL-C have been proved to be significantly associated with increased risks of hemorrhagic stroke (HS) and CMBs (17, 19, 24). Thus, the risk of hemorrhagic events appears to depend on the reduction extent and degree of blood lipid levels rather than on statin use *per se* (17–22, 24). Unfortunately, the current international guidelines on lipid modification are all committed to emphasize the intensive reduction for LDL-C to prevent ischemic events, with no instructions on a more propriate target range to balance the prevention of ischemic stroke recurrence and the prevention of hemorrhagic events, especially for patients requiring high-dose statin treatment. Therefore, it is of great scientific and practical significance to conduct prospective studies to reveal the clinical and neuroimaging characteristics of CMBs in acute ischemic stroke (AIS) patients, and to explore the correlation between intensive lipid-lowering therapy and hemorrhage risk.

## Methods

### Study design

The CHRISTMAS trial is a multicenter, prospective, randomized controlled trial design. Participants will be enrolled in five hospitals from two provinces in China (Sichuan and Chongqing). This investigator-initiated clinical trial will be conducted in adherence to the Good Clinical Practice guidelines and in compliance with the Declaration of Helsinki, including all revisions. The study has been approved by the ethics committee of Sichuan Academy of Medical Sciences & Sichuan Provincial People's Hospital (SAMS & SPPH) and all participating centers, and has been registered at Clinicaltrials.gov with the identifier: NCT05589454 (Supplementary material 1). Written informed consent from eligible patients or their legally authorized representatives will be obtained before patient enrollment.

### Participants

By December 2023, 344 patients will have been enrolled. The inclusion and exclusion criteria are provided in Table 1. Briefly, patients are eligible if they (1) have a non-cardioembolic ischemic stroke within 14 days from the onset of the symptoms prior to the study participation; (2) are between the ages of 18 and 90; and (3) have the presence of CMBs (without differentiating the number or localization) on baseline susceptibility-weighted imaging (SWI) (Supplementary material 2). Key exclusion criteria include active hemorrhagic diseases, hemorrhagic tendency, severe stroke, or other severe conditions that are not eligible for clinical trials. In addition, patients who have contraindications to statins or need for intravenous thrombolysis, interventional therapy, anticoagulation, or dual antiplatelet therapy shall be excluded.

**Table 1:** Inclusion and exclusion criteria.

**Inclusion criteria**
1. Patients with a non-cardioembolic ischemic stroke within 14 days prior to entry to the study.
2. Adults between the ages of 18 and 90.
3. Patients with CMBs on baseline SWI imaging.
4. Patients or their legal representatives volunteer to participate and sign written informed consent.
**Exclusion criteria**
1. Patients with severe ASI (NIHSS score ≥21).
2. Patients with coma (GCS score < 8).
3. Patients with previous moderate to severe dependence (mRS score 3–5).
4. Patients with any contraindications to CT and MRI (such as metal implants, claustrophobia, etc.).
5. Patients who are allergic to atorvastatin or excipients.
6. Patients with intracranial hemorrhagic diseases confirmed by CT or MRI, such as cerebral hemorrhage, epidural hematoma, subdural hematoma, ventricular hemorrhage, subarachnoid hemorrhage, traumatic cerebral hemorrhage, or hemorrhagic conversion of infarcts, etc.
7. Patients within 6 months after hemorrhagic stroke.
8. Patients with hemorrhagic tendency, such as abnormal coagulation function, Henoch-Schonlein purpura, platelet count < 100 × 10^9^/L or abnormal platelet function, etc.
9. Patients who are ready to undergo or have undergone intravenous thrombolysis after the onset of the disease or who require urgent or recent (within 90 days) endovascular treatment.
10. Patients with severe hypertension (systolic blood pressure ≥185 mmHg or diastolic blood pressure ≥110 mmHg) that has not been controlled by treatment.
11. Patients with hypoglycemia (< 2.7 mmol/L) or hyperglycemia (>22.2 mmol/L).
12. Patients with previous cerebral arteritis, brain tumor, cerebral parasitic disease, cerebral arteriovenous malformation, cerebral cavernous hemangioma, cerebral aneurysm, severe craniocerebral injury, or intracranial infection.
13. Patients with previous severe valvular heart disease, atrial fibrillation, acute myocardial infarction, or interventional therapy in the past 6 months, heart failure (patients classified as class III-IV according to the NYHA) or patients with indications for pacemaker placement but without pacemaker installation or other malignant arrhythmias.
14. Patients contraindicate to antiplatelet therapy.
15. Patients who must use other types of statins or other types of lipid-lowering drugs such as ezetimibe.
16. Patients with severe mental disorders, or dementia that are unable or unwilling to cooperate.
17. Patients with active liver disease or unexplained two or more abnormal liver function tests [(ALT) or (AST) ≥ 3.0 × (ULN)].
18. Patients with myositis, myopathy, rhabdomyolysis, or two or more episodes of unexplained serum [CK] elevation [(CK) ≥ 5.0 × ULN].
19. Patients with other serious systemic or organic diseases that investigators believe will not allow evaluation of efficacy or are unlikely to complete the expected course of treatment and follow-up (e.g., malignancy, life expectancy < 3 years, etc.).
20. Women who are pregnant, breastfeeding, or planning to become pregnant and who do not want to use contraception.
21. Patients who enrolled in other clinical trials during the 3 months prior to the study.
22. Patients deemed ineligible for clinical trial participation by the investigator.
23. Patients or their legal representatives do not consent to participate in this study.

AIS, acute ischemic stroke; ALT, alanine aminotransferase; AST, aspartate aminotransferase; CK, creatine kinase; CT, computed tomography; CMBs, cerebral microbleeds; GCS, Glasgow Coma Scale; mRS, modified Rankin Scale; NIHSS, National Institutes of Health Stroke Scale; NYHA, New York Heart Association; SWI, susceptibility weighted imaging; TIA, transient ischemic attack; ULN, upper limit of normal.

### Randomization and blinding

**Figure 1:**
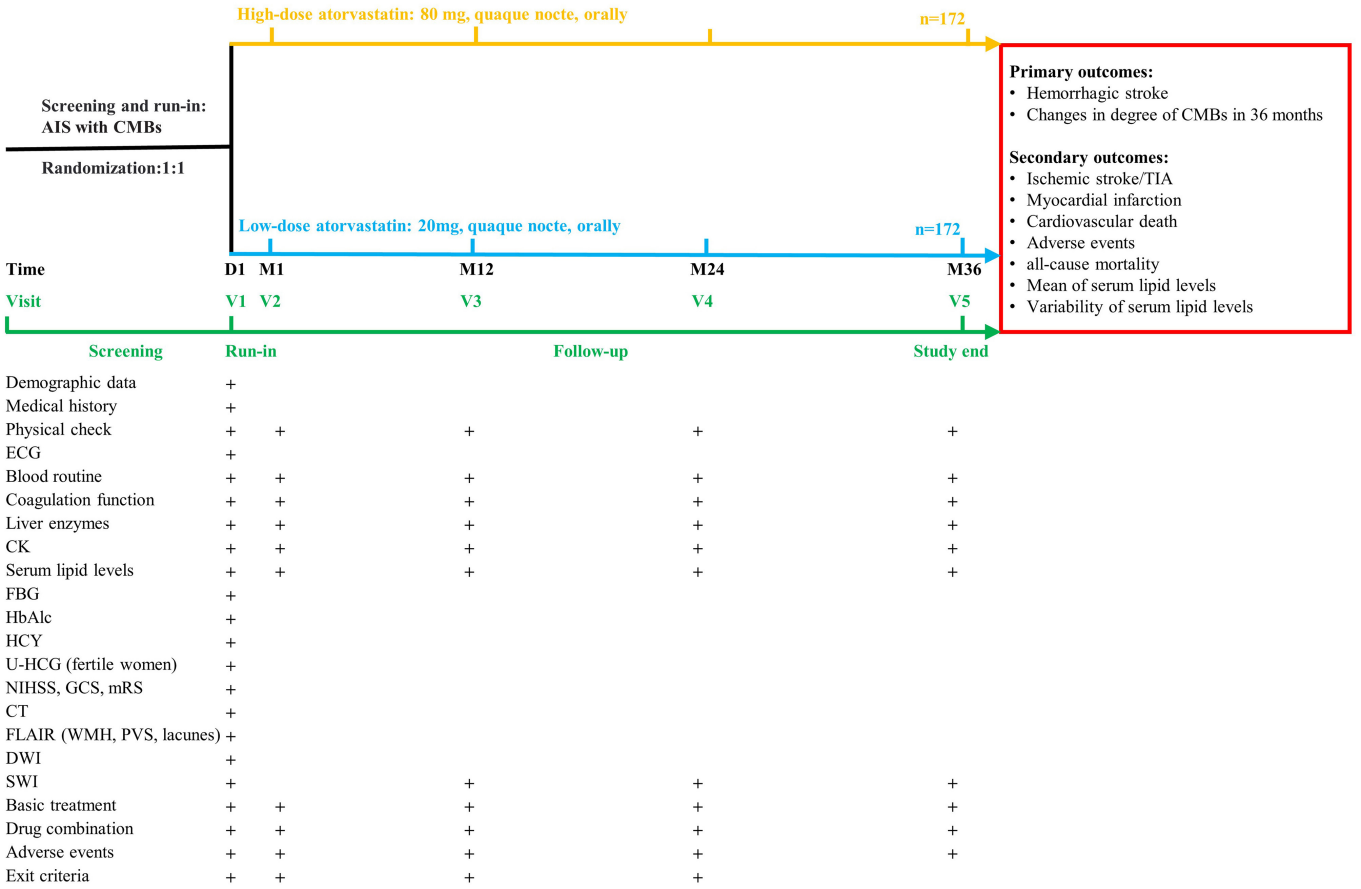
Design of the CHRISTMAS trial. AIS, acute ischemic stroke; CK, creatine kinase; CT, computed tomography; CMBs, cerebral microbleeds; DWI, diffusion weighted imaging; ECG, electrocardiograph; FBG, fasting blood-glucose; FLAIR, fluid-attenuated inversion-recovery sequency; GCS, Glasgow Coma Scale; mRS, modified Rankin Scale; HbAlc, glycosylated hemoglobin; HCY, homocysteine; NIHSS, National Institutes of Health Stroke Scale; PVS, perivascular spaces; SWI, susceptibility weighted imaging; TIA, transient ischemic attack; U-HCG, urine human chorionic gonadotropin; ULN, upper limit of normal; WMH, white matter hyperintensities.

All patients who are undergoing an AIS will be screened timely according to inclusion and exclusion criteria by a qualified investigator of each center. Then the eligible patients will be randomized into high-dose and low-dose atorvastatin arms in a 1:1 ratio (Figure 1). The concealment of allocation will be performed using sealed envelopes with numbers by the participants' physicians. Considering the non-subjective nature of the primary outcomes, as well as the reason that the investigator-initiated clinical trial without technical and financial support from any pharmaceutical manufacturer to develop placebos that meet the study design requirements in clinical practice, the study finally gave up the design and application of placebo, and only designed a single-blind manner for the study investigators. So, it's important to note that neither the participants nor their physicians conducting the drug intervention can be blind to the grouping, because the number of tablets administered in different groups will be different, so these physicians cannot be involved in the research process. All investigators and data collectors will not participate in the whole intervention.

### Treatment

- High-dose atorvastatin arm: Atorvastatin calcium tablets four pills (80 mg), quaque nocte (24 ± 1 h between two doses), orally, until the end of follow-up.
- Low-dose atorvastatin arm: Atorvastatin calcium tablets one pill (20 mg), quaque nocte (24 ± 1 h between two doses), orally, until the end of follow-up.

It is strongly recommended in both arms for the optimal treatment including acute treatment and other secondary prevention agents in accordance with clinical practice guidelines and the best medical conditions in the health care unit.

### Follow-up

Detailed follow-up schedules and procedures are provided in Figure 1. Baseline characteristics, SWI and clinical laboratory tests will be obtained at enrollment. The following assessments will be performed at 1 month and annually follow-up: blood tests, SWI, study compliance, concomitant medication, adverse events, and withdrawal criteria. As it is not clear whether participants in this study would benefit more from following current guidelines or moderately relaxed lipid management targets. No further interventions which might violate the study protocol should be taken (such as suggesting withdrawal from the study or adjusting the treatment plan, etc.) if it is found that the serum lipid levels of the participants do not meet the current guidelines either for both groups. But the participants' doctor should be informed to strictly monitor their lipid levels and clinical conditions.

### Outcomes

#### Primary outcomes

The trial has co-primary outcome, namely, hemorrhage risk.

The first part is the incidence of HS confirmed by brain computed tomography (CT) or magnetic resonance imaging (MRI).

The second part is the changes in degree of CMBs until the end of 36 month follow-up.

#### Secondary outcomes

The incidence of recurrent IS and TIA.

The incidence of myocardial infarction.

The incidence of cardiovascular death.

The incidence of adverse events.

The incidence of all-cause mortality.

The mean of serum lipid levels [TG, TC, LDL-C, and high-density lipoprotein cholesterol (HDL-C)] for 36 months with at least 3 measurements.

The variability of serum lipid levels (TG, TC, LDL-C, and HDL-C) for 36 months with at least 3 measurements.

### Sample size estimates

Sample size calculations were based on the primary outcomes (the incidence of ICH). All participants are expected to be enrolled for 1 year and followed for 36 months. It will be tested whether the high-dose statin arm suffers a higher risk of hemorrhage than the low-dose statin arm in terms of the primary outcomes for 36 months. According to the SPARCL trial (the incidence of ICH: 25.2 vs. 12.0% in high-dose statin arm vs. low-dose statin arm with 4.9 years of mean follow-up duration) (17). The sample size was calculated by PASS software (version 15.0) with a two-sided type I error of 5% (α = 0.05) and 80% power, a sample size of 137 patients in each arm is required (total 274 patients). Assuming around 20% loss to follow-up, a total of 344 patients will be randomized.

### Statistical analysis

SPSS 25.0 software will be used for statistical analysis by data analysts who are blind to the study. Two-sided *P*-values will be calculated with a significant level of 5%, and the parameters will be estimated by 95% confidence intervals. The primary analysis will be under intention-to-treat (ITT) principle and be performed on both the ITT population and per-protocol (PP) population.

Descriptive statistics of demographic data and other baseline eigenvalues will be performed on the ITT population for both groups. Continuous variables will be presented as mean (SD) or median (IQR). Categorical variables will be presented as numbers (%). For continuous variables and categorical variables, *t*-test/rank sum test and chi-square test/Fisher exact test will be used to detect possible disequilibrium between groups.

The outcomes will be performed on both ITT and PP population. Initially, the number and percentage of HS cases and patients with mild-to-severe CMBs at baseline and annul follow-up will be calculated and compared between the two arms by Chi-square test or Fisher's exact test. Then, for all time-to-event models, cumulative event rate during the follow-up will be estimated using the Kaplan-Meier method and will be compared with the log-rank test. If needed, Cox proportional hazard regression analysis will be used after testing proportional hazard assumption and important potential covariates will be adjusted for sensitivity analyses. The mean and variability of serum lipid levels in 36 month follow-up with at least three measurements will be calculated and compared by Student *t*-test or Wilcoxon rank sum test. Then generalized linear mixed model will be used to analyze the correlation between the proportion of CMBs with different degrees and serum lipid levels over time.

The interim analysis will be performed when 172 subjects have completed at least one follow-up SWI, and it will be focused on subject recruitment, comparability of baseline between groups, sample size assumptions to account for event incidence, follow-up shedding rate, adverse event data, and the effect of treatment on the primary and secondary outcomes.

## Discussion

CMBs have been identified as a potential neuroimaging biomarker of SCVD that are prone to spontaneous ICH (10). In IS cohorts, CMBs were found to be associated with the risks of both subsequent ICH and recurrent IS, while the relative risk of subsequent ICH seemed to rise more steeply than that of IS as the burden of CMBs increased (13, 14). This suggests that in the secondary prevention of IS, we need to pay attention to the risk of hemorrhagic events in addition to the benefit of ischemic events, especially for patients with CMBs.

At present, as a choice for secondary prevention of arteriosclerotic cardiovascular disease (ASCVD) patients and primary prevention of patients with elevated risk of ASCVD, statins have been widely used in clinical practice. The SPARCL study was the first to demonstrate that 80 mg of atorvastatin per day reduced the overall incidence of strokes and cardiovascular events in patients with recent stroke or TIA and without known coronary heart disease (17). But this study also revealed an increased risk of ICH related to high-dose statins (17). A recent meta-analysis showed that serum levels of major lipids (TC, TG, and LDL) were negatively correlated with CMBs, but there was no sufficient evidence for statin use as a risk factor for CMBs (19). Thus, it is reasonable to speculate that the risk of statin-related hemorrhage appears to depend on the extent to which certain serum lipid levels are reduced, rather than on statin use *per se*. Taken together, these findings present a paradoxical problem: high-dose statins have a protective effect on IS recurrence but are likely to be harmful for hemorrhage risk, including HS and CMBs.

In addition, in the secondary prevention of IS, national guidelines around the world delineate different lipid regulation criteria for different baseline conditions, emphasizing the use of high-intensity or maximum tolerated doses of statins to achieve serum LDL-C < 1.8 mmol/L (70 mg/dl) and/or a ≥50% reduction from baseline in those most likely to benefit (25, 26). However, current clinical studies and practice guidelines have failed to suggest lower limits for lipid management goals. Several studies have confirmed that lower serum TG, TC, and LDL-C levels increased the risk of CMBs (17, 19, 24, 27), and the presence of CMBs was associated with increased subsequent ICH risk and poor functional prognosis in patients with AIS (28). These studies suggest that unlimited reduction of serum major lipid levels, mainly LDL-C, is likely to be detrimental in the secondary prevention of IS, especially in IS with CMBs. Therefore, further studies are urgently needed to explore the safe lower limits of lipid management goals in IS with CMBs.

## Conclusions

In conclusion, the CHRISTMAS trial is the first and largest secondary prevention trial about lipid-lowering therapy for patients at high risk of future HS. Our results will inform clinical decisions regarding the long-term lipid management in these patients, which is a growing dilemma in clinical practice.

## Ethics statement

The studies involving human participants were reviewed and approved by Ethics Committee of Sichuan Academy of Medical Sciences and Sichuan Provincial People's Hospital. The patients/participants provided their written informed consent to participate in this study.

## Author contributions

J-lZ and YX designed the study and revised the manuscript. J-lZ, WY, JT, LZ, YL, and SG drafted the protocol. YX, C-bA, LW, S-jY, JW, and T-qY coordinated the trial. All authors contributed to the article and approved the submitted manuscript.
