# Supplementary material for: A multicenter, prospective, randomized controlled trial of intracranial hemorrhage risk of intensive statin therapy in patients with acute ischemic stroke combined with cerebral microbleeds (CHRISTMAS): Study protocol
Source: Front Neurol. 2023 Feb 9;14:1097078. doi: 10.3389/fneur.2023.1097078 (PMC9948086; doi:10.3389/fneur.2023.1097078)
Supplement: Supplementary file 1 [file Data_Sheet_1.PDF]

**ClinicalTrials.gov Protocol Registration and Results System (PRS) Receipt**
Release Date: October 19, 2022

**ClinicalTrials.gov ID: NCT05589454**

## Study Identification

Unique Protocol ID: zjl8803302022NSCSC1374

Brief Title: Intracranial Hemorrhage Risk of Intensive Statin in Acute Ischemic Stroke With Cerebral Microbleeds

Official Title: Intracranial Hemorrhage Risk of Intensive Statin Therapy in Patients With Acute Ischemic Stroke Combined With Cerebral Microbleeds

Secondary IDs:

## Study Status

Record Verification: October 2022

Overall Status: Not yet recruiting

Study Start: January 2023 [Anticipated]

Primary Completion: December 2026 [Anticipated]

Study Completion: June 2027 [Anticipated]

## Sponsor/Collaborators

Sponsor: Sichuan Provincial People's Hospital

Responsible Party: Principal Investigator
Investigator: Jialing Zhao [jzhao]
Official Title: Principal Investigator
Affiliation: Sichuan Provincial People's Hospital

Collaborators: Science & Technology Department of Sichuan Province

## Oversight

U.S. FDA-regulated Drug: No

U.S. FDA-regulated Device: No

U.S. FDA IND/IDE: No

Human Subjects Review: Board Status: Pending
Board Name: Ethics Committee
Board Affiliation: Sichuan Academy of Medical Sciences & Sichuan Provincial People's Hospital
Phone: 02887393999
Email: jailynyy@163.com
Address:

32# W. Sec 2, 1st Ring Rd, Qingyang District, Chengdu City, Sichuan Province, China

Data Monitoring: No

FDA Regulated Intervention: No

## Study Description

Brief Summary: This study is the first and largest secondary prevention trial about lipid-lowering therapy for acute ischemic stroke patients at high-risk of intracranial hemorrhage.

The primary hypothesis of this study is: excessive reduction in serum lipid levels by intensive statin therapy in acute ischemic stroke patients with cerebral microbleeds can increase the risk of intracranial hemorrhage.

This study will shed light on new clinical decisions regarding the long-term serum lipid management in these patients with dilemma in clinical practice.

Detailed Description: Cerebral microbleeds are an important subtype of cerebral small vessel diseases that have been established in approximately one third of patients with ischemic stroke and are associated with the risk of recurrent ischemic stroke, symptomatic intracranial hemorrhage, and all-cause death. In patients with ischemic stroke or transient ischemic attack, the relative and absolute risks of intracranial hemorrhage increase more rapidly than the risk of ischemic stroke with the increase of cerebral microbleeds burden, but the absolute incidence of ischemic stroke is still higher than that of cerebral hemorrhage.

It has been generally accepted that statins can effectively prevent recurrent ischemic stroke by reducing serum lipid levels. However, both low serum lipid levels and high dose of statins are clear risk factors for intracerebral hemorrhage, and the reduction of major serum lipid levels may increase the risk of cerebral microbleeds. Of note, the risk of statin mediated hemorrhage appears to depend on the degree of lipid reduction rather than statin use per se. These observations raise concerns about the safety of lipid-lowering therapy, especially intensive lipid-lowering therapy, in patients with acute ischemic stroke and cerebral microbleeds who are at high risk for future intracranial hemorrhage. It is still not clear that how to carry on the proper management of serum lipid levels in this particular population to reduce the recurrence of ischemic events as well as hemorrhagic events, for there is still a lack of clinical studies to explore the risk and benefit of different doses of statins to achieve different degrees of lipid regulation.

So, if it is proved that excessive reduction in serum lipid levels by intensive statin therapy in acute ischemic stroke patients with cerebral microbleeds can increase the risk of future intracranial hemorrhage, we will inform new clinical decisions regarding the long-term lipid management in these patients with dilemma in clinical practice.

## Conditions

Conditions: Acute Ischemic Stroke
Cerebral Microbleeds

Keywords: Acute Ischemic Stroke
Intracranial Hemorrhage Risk
Cerebral Microbleeds
Intensive Statin Therapy

## Study Design

| | |
|---|---|
| Study Type: | Interventional |
| Primary Purpose: | Prevention |
| Study Phase: | Phase 4 |
| Interventional Study Model: | Parallel Assignment |
| Number of Arms: | 2 |
| Masking: | None (Open Label) |
| Allocation: | Randomized |
| Enrollment: | 344 [Anticipated] |

## Arms and Interventions

| Arms | Assigned Interventions |
|---|---|
| Experimental: High-dose atorvastatin<br>atorvastatin calcium tablets 80 mg, quaque nocte, continue to the end of the study | Drug: Atorvastatin Calcium tablets 80mg<br>Atorvastatin calcium tablets 4 pills (80 mg) will be given at a fixed time every night (24 ± 1 h between two doses) , orally, until the end of follow-up<br>Other Names:<br>• ALe produced by Jialin pharmaceutical company |
| Active Comparator: Low-dose atorvastatin<br>atorvastatin calcium tablets 20 mg, quaque nocte, continue to the end of the study | Drug: Atorvastatin Calcium tablets 20mg<br>Atorvastatin calcium tablets 1 pill (20 mg) will be given at a fixed time every night (24 ± 1 h between two doses) , orally, until the end of follow-up<br>Other Names:<br>• ALe produced by Jialin pharmaceutical company |

## Outcome Measures

Primary Outcome Measure:

1. The incidence of hemorrhagic strokes
   [Time Frame: From date of randomization until the date of the first occurrence of hemorrhagic stroke, assessed up to 36 months]
2. Changes in degree of cerebral microbleeds
   The degree is divided into: mild (1-2), moderate (3-10), severe (more than 10), calculate and compare the proportions of different degrees at baseline and the end of the study

   [Time Frame: From date of randomization until the end of the study, assessed up to 36 months]

Secondary Outcome Measure:

3. The Incidence of recurrent ischemic stroke and transient ischemic attack
   [Time Frame: From date of randomization until the date of the first recurrent of ischemic stroke or the first occurrence of transient ischemic attack, assessed up to 36 months]
4. The Incidence of myocardial infarction
   [Time Frame: From date of randomization until the date of the first occurrence of myocardial infarction, assessed up to 36 months]
5. The Incidence of cardiovascular death
   [Time Frame: From date of randomization until the date of cardiovascular death, assessed up to 36 months]
6. The mean of serum triglycerides (TG) levels
   Calculate the mean of serum TG levels for 3 years with at least 3 measurements

[Time Frame: From date of randomization until the end of the study, assessed up to 36 months]

7. The mean of serum total cholesterol (TC) levels
   Calculate the mean of serum TC levels for 3 years with at least 3 measurements

   [Time Frame: From date of randomization until the end of the study, assessed up to 36 months]

8. The mean of serum low-density lipoprotein cholesterol (LDL-C) levels
   Calculate the mean of serum LDL-C levels for 3 years with at least 3 measurements

   [Time Frame: From date of randomization until the end of the study, assessed up to 36 months]

9. The mean of serum high-density lipoprotein cholesterol (HDL-C) levels
   Calculate the mean of serum HDL-C levels for 3 years with at least 3 measurements

   [Time Frame: From date of randomization until the end of the study, assessed up to 36 months]

10. The variability of serum triglycerides (TG) levels
   Calculate the variability of serum TG levels for 3 years with at least 3 measurements

   [Time Frame: From date of randomization until the end of the study, assessed up to 36 months]

11. The variability of serum total cholesterol (TC) levels
   Calculate the variability of serum TC levels for 3 years with at least 3 measurements

   [Time Frame: From date of randomization until the end of the study, assessed up to 36 months]

12. The variability of serum low-density lipoprotein cholesterol (LDL-C) levels
   Calculate the variability of serum LDL-C levels for 3 years with at least 3 measurements

   [Time Frame: From date of randomization until the end of the study, assessed up to 36 months]

13. The variability of serum high-density lipoprotein cholesterol (HDL-C) levels
   Calculate the variability of serum HDL-C levels for 3 years with at least 3 measurements

   [Time Frame: From date of randomization until the end of the study, assessed up to 36 months]

14. The proportions of different degrees of CMBs at the end of the study
   Calculate the proportions of different degrees of CMBs (mild, moderate and severe) at the end of the study

   [Time Frame: From date of randomization until the end of the study, assessed up to 36 months]

15. The correlation between the mean of serum triglycerides (TG) levels and the proportions of different degrees of CMBs
   [Time Frame: From date of randomization until the end of the study, assessed up to 36 months]

16. The correlation between the mean of serum total cholesterol (TC) levels and the proportions of different degrees of CMBs
   [Time Frame: From date of randomization until the end of the study, assessed up to 36 months]

17. The correlation between the mean of serum low-density lipoprotein cholesterol (LDL-C) levels and the proportions of different degrees of CMBs
   [Time Frame: From date of randomization until the end of the study, assessed up to 36 months]

18. The correlation between the mean of serum high-density lipoprotein cholesterol (HDL-C) levels and the proportions of different degrees of CMBs
   [Time Frame: From date of randomization until the end of the study, assessed up to 36 months]

19. The correlation between the variability of serum triglycerides (TG) levels and the proportions of different degrees of CMBs
   [Time Frame: From date of randomization until the end of the study, assessed up to 36 months]

20. The correlation between the variability of serum total cholesterol (TC) levels and the proportions of different degrees of CMBs
   [Time Frame: From date of randomization until the end of the study, assessed up to 36 months]

21. The correlation between the variability of serum low-density lipoprotein cholesterol (LDL-C) levels and the proportions of different degrees of CMBs
   [Time Frame: From date of randomization until the end of the study, assessed up to 36 months]

22. The correlation between the variability of serum high-density lipoprotein cholesterol (HDL-C) levels and the proportions of different degrees of CMBs

[Time Frame: From date of randomization until the end of the study, assessed up to 36 months]

## Eligibility

Minimum Age: 18 Years

Maximum Age: 85 Years

Sex: All

Gender Based: No

Accepts Healthy Volunteers: No

Criteria: Inclusion Criteria:

1. Patients with a non-cardioembolic ischemic stroke within 14 days prior to entry to the study
2. Adults between the ages of 18 and 85
3. Patients with cerebral microbleeds on baseline SWI imaging
4. Patients or their legal representatives volunteer to participate and sign written informed consent

Exclusion Criteria:

1. Patients with severe acute ischemic stroke (NIHSS score ≥21)
2. Patients with coma (GCS score < 8)
3. Patients with previous moderate to severe dependence (mRS score 3-5)
4. Patients with any contraindications to CT and MRI (such as metal implants, claustrophobia, etc.)
5. Patients who are allergic to atorvastatin or excipients
6. Patients with intracranial hemorrhagic diseases confirmed by CT or MRI, such as cerebral hemorrhage, epidural hematoma, subdural hematoma, ventricular hemorrhage, subarachnoid hemorrhage, traumatic cerebral hemorrhage or hemorrhagic conversion of infarcts, etc
7. Patients within 6 months after hemorrhagic stroke
8. Patients with hemorrhagic tendency, such as abnormal coagulation function, Henoch-Schonlein purpura, platelet count less than 100×109/L or abnormal platelet function, etc
9. Patients who are ready to undergo or have undergone intravenous thrombolysis after the onset of the disease or who require urgent or recent (within 90 days) endovascular treatment;
10. Patients with severe hypertension (systolic blood pressure ≥ 185 mmHg or diastolic blood pressure ≥ 110 mmHg) that has not been controlled by treatment
11. Patients with hypoglycemia (< 2.7 mmol/L) or hyperglycemia (#22.2 mmol/L)
12. Patients with previous cerebral arteritis, brain tumor, cerebral parasitic disease, cerebral arteriovenous malformation, cerebral cavernous hemangioma, cerebral aneurysm, severe craniocerebral injury, or intracranial infection
13. Patients with previous severe valvular heart disease, atrial fibrillation, acute myocardial infarction or interventional therapy in the past 6 months, heart failure (patients classified as class III-IV according to the New York Heart Association [NYHA]) or patients with indications for pacemaker placement but without pacemaker installation or other malignant arrhythmias
14. Patients contraindicate to antiplatelet therapy;
15. Patients who must use other types of statins or other types of lipid-lowering drugs such as ezetimibe

16. Patients with severe mental disorders or dementia that are unable or unwilling to cooperate
17. Patients with active liver disease or unexplained 2 or more abnormal liver function tests (alanine aminotransferase [ALT] or aspartate aminotransferase [AST] ≥ 3.0× upper limit of normal [ULN])
18. Patients with myositis, myopathy, rhabdomyolysis, or 2 or more episodes of unexplained serum creatine kinase[CK] elevation ([CK]≥5.0×ULN)
19. Patients with other serious systemic or organic diseases that investigators believe will not allow evaluation of efficacy or are unlikely to complete the expected course of treatment and follow-up (e.g., malignancy, life expectancy < 3 years, etc.)
20. Women who are pregnant, breastfeeding or planning to become pregnant and who do not want to use contraception
21. Patients who participated in or are participating in other clinical trials during the 3 months prior to the study
22. Patients who are deemed ineligible for clinical trial participation by the investigator
23. Patients or their legal representatives do not consent to participate in this study

## Contacts/Locations

Central Contact Person: Jialing Zhao, MD
Telephone: +8618113137196
Email: jailynyy@163.com

Central Contact Backup:

Study Officials: Jialing Zhao, MD
Study Principal Investigator
Departement of Neurology, Sichuan Academy of Medical Sciences & Sichuan Provincial People's Hospital

Yang Xiang, MD
Study Director
Departement of Neurology, Sichuan Academy of Medical Sciences & Sichuan Provincial People's Hospital

Locations: **China, Sichuan**
Sichuan Academy of Medical Sciences & Sichuan Provincial People's Hospital
Chengdu, Sichuan, China, 610072

Zigong Third People's Hospital
Zigong, Sichuan, China, 643021

**China, Chongqing**
Yunyang County People's Hospital
Chongqing, Chongqing, China, 404599

**China, Sichuan**
Chengdu Eighth People's Hospital
Chengdu, Sichuan, China, 610017

Ya 'an People's Hospital
Ya'an, Sichuan, China, 625000

## IPDSharing

Plan to Share IPD: No

## References

Citations:

Links:

Available IPD/Information:
