# Supplementary material for: A multicenter, prospective, randomized controlled trial of intracranial hemorrhage risk of intensive statin therapy in patients with acute ischemic stroke combined with cerebral microbleeds (CHRISTMAS): Study protocol
Source: Front Neurol. 2023 Feb 9;14:1097078. doi: 10.3389/fneur.2023.1097078 (PMC9948086; doi:10.3389/fneur.2023.1097078)
Supplement: Supplementary file 2 [file Data_Sheet_2.DOCX]

**Magnetic Resonance Imaging acquisition and analysis**

All magnetic resonance imaging (MRI) assessments will be performed on 1.5-T or 3.0-T scanners utilizing standard T1-weighted 3D magnetization-prepared rapid gradient echo (repetition time (TR)/echo time (TE) =1900/7 ms and slice thickness = 6 mm) sequences, T2-weighted 3D fast spin-echo (TR/TE = 4600/115 ms and slice thickness = 6 mm) sequences , axial fluid-attenuated inversion recovery (TR/TE = 7600/125 ms and slice thickness = 6 mm) sequences, diffusion weighted imaging (TR/TE = 3800/90 ms and slice thickness = 6 mm) and susceptibility weighted imaging (TR/TE = 28/18 ms and slice thickness = 1.6 mm) sequences. Imaging data will be collected in digital imaging and communications in medicine (DICOM) format on discs and further analyzed by two designated neuroradiologists blinded to clinical information.

**Radiological assessment of CMBs, WMH, PVS and lacunes**

Subtypes of cerebral small vessel disease are determined in accordance with Standards for ReportIng Vascular changes on nEuroimaging (STRIVE). Cerebral microbleeds (CMBs) are visualized as small (generally 2-5 mm in diameter, but up to 10 mm) areas of signal void with associated blooming seen on T2*-weighted MRI or other sequences that are sensitive to susceptibility effects and are generally not seen on CT, or on FLAIR, T1-weighted, or T2-weighted sequences. White matter hyperintensities (WMH) of presumed vascular origin are hyperintense on T2-weighted or FLAIR sequences but can appear as isointense or hypointense (although less hypointense than cerebrospinal fluid (CSF)) on T1-weighted sequences, depending on the sequence parameters and the severity of the pathological changes, while lesions in the subcortical grey matter or brainstem are not included in this category. Perivascular spaces (PVS) are defined as fluid-filled spaces that follow the typical course of a vessel as it goes through grey or white matter. The spaces have signal intensity similar to that of CSF on all sequences; because they follow the course of penetrating vessels, they appear linear when imaged parallel to the course of the vessel, and round or ovoid, with a diameter generally smaller than 3 mm, when imaged perpendicular to the course of the vessel. Lacunes are defined as cavities with diameters of 3-15 mm with cerebrospinal-fluid-like signal intensity on a combination of T1-weighted, T2-weighted, and FLAIR images, consistent with a previous acute small deep brain infarct or hemorrhage in the territory of one perforating arteriole.

**Fazekas Visual Rating Scale**

The degree of WMH severity will be rated visually on axial FLAIR images using the modified Fazekas scale. This scale divides WMHs into periventricular and deep sites to rate separately. Periventricular WMHs (pWMHs) are scored as follows: 0 = absence, 1 = caps or pencil-thin lining, 2 = smooth halo, and 3 = irregular periventricular hyperintensities extending into the deep white matter. Deep WMHs (dWMHs) are scored as follows: 0 = absence, 1 = punctuate foci, 2 = beginning confluence of foci, and 3 = large confluent areas. The total score (0 to 6) will be calculated by adding the scores of pWMHs and dWMHs.

**Quantitative analysis of WMH volume**

All scans will be inspected visually, and WMH volumes will be quantified using the software programs MRICRON (Version 1.9.1, [www.nitrc.org/projects/mricro](http://www.nitrc.org/projects/mricro) accessed on March of 2021) and ITK-SNAP (Version 4.0.0, [www.itksnap.org](http://www.itksnap.org) accessed on March of 2022). The MRICRON software will be used to extract the effective WMH area, then the ITK-SNAP software will be used to calculate the WMH volume.
